# One healthy live birth after preimplantation genetic testing of a cryptic balanced translocation (9;13) in a family with cerebral palsy and glaucoma: a case report

**DOI:** 10.1186/s12920-021-00938-7

**Published:** 2021-03-17

**Authors:** Xiliang Wang, Changsheng Wu, Dongmei Hao, Jinyan Zhang, Chang Tan, De-hua Cheng, Jia Fei, Yuexin Yu

**Affiliations:** 1 Department of Reproductive Medicine, General Hospital of Northern Theater Command, Shenyang, China; 2 Peking Jabrehoo Med Tech Co., Ltd, Beijing, China; 3 Reproductive and Genetic Hospital of CITIC-Xiangya, Changsha, Hunan China

**Keywords:** Cryptic balanced translocation, G-banding, FISH, Preimplantation genetic testing

## Abstract

**Background:**

Cryptic balanced translocations often evade detection by conventional cytogenetics. The preimplantation genetic testing (PGT) technique can be used to help carriers of balanced translocations give birth to healthy offspring; however, for carriers of cryptic balanced translocations, there is only one report about trying assisted reproduction using the PGT technique but with no pregnancy.

**Case presentation:**

A couple had 3 births out of 4 pregnancies, and all died very young, with two of them having both cerebral palsy and glaucoma. The husband with oligoasthenospermia was found to be a cryptic balanced translocation carrier for t (9,13) (p24.3, q31.3) with G-banding, FISH (fluorescence in-situ hybridization), and MicroSeq techniques; live birth of a healthy baby girl was achieved with PGT/NGS (next-generation sequencing) for the couple.

**Conclusion:**

Here, we report for the first time a successful live birth of a healthy baby through the PGT technique for a family in which the husband is a carrier of the cryptic balanced translocation t (9,13) (p24.3, q31.3), presumably causative for cerebral palsy and glaucoma. Our study showed that the PGT/NGS technique can effectively help families with a cryptic balanced translocation have healthy offspring.

## Background

Balanced translocations, with no copy number variations, are the most common kind of chromosomal structural aberrations in humans, with an incidence of balanced translocation in newborn infants of approximately 1/500–1/625 [1, 2]. The carriers of balanced translocations generally have no phenotypic abnormality but have a high chance of producing unbalanced gametes when forming germ cells that eventually lead to spontaneous abortion or birth defects.

There are several methods for detecting balanced translocations, including G-banding karyotyping, FISH (fluorescence in-situ hybridization), array CGH (array comparative genomic hybridization) and NGS (next-generation sequencing). G-banding karyotyping has a limit of detection of genomic imbalances above 5–10 Mb [3]; FISH or array-based methods can detect chromosomal abnormalities down to 100 kb [4]; NGS technology has an even higher resolution, although with relatively deeper sequencing depth [5], and even has the benefit of improved breakpoint resolution [6]. Thus, a normal result obtained after G-banding karyotyping may actually be revealed as a cryptic balanced translocation after high-resolution karyotyping, especially in cases involving subtelomeric regions, which often escape detection by traditional G-banding techniques [7].

PGT (preimplantation genetic testing) evolved from PGT-A for the analysis of aneuploidies to PGT-M (monogenic disease), up to PGT-SR (structural rearrangements) [8], to answer to the growing request of more accurate genetic diagnosis of eventual smaller genetic defects. However, different countries allow different approach to these tests, since legal position of the embryo itself may vary [9]. It has long been known that preimplantation genetic testing can be used to help carriers of balanced translocations give birth to unaffected offspring; however, for carriers of cryptic balanced translocations, there is only one report of a successful PGT with 4 normal embryos out of 18, which however did not lead to a successful pregnancy [10], and this may be associated with the significantly lower incidence of balanced or normal gametes for balanced translocations with terminal breakpoints than those without terminal breakpoints [11].

Here, we report the first successful achievement of a live birth of a healthy baby through the PGT technique for a carrier of the cryptic balanced translocation of t (9,13) (p24.3, q31.3).

## Case presentation

**Fig. 1:**
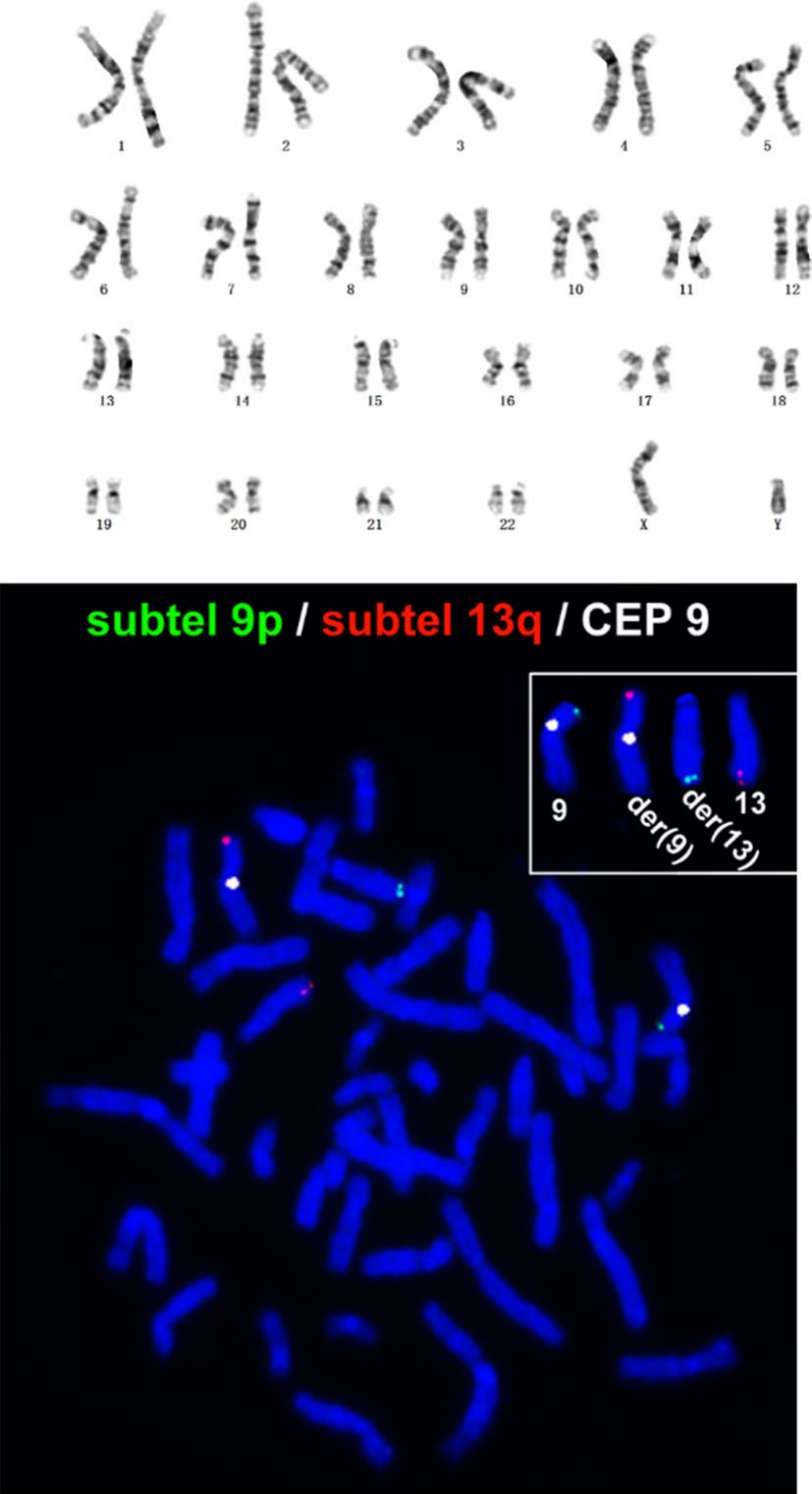
Karyotyping and FISH results of the husband. **a** G-banding karyotype of the father: normal (upper); **b** FISH results of the father: t (9;13) (p24.3; q31.3) (lower)

After 3 early deaths of their very young children, 2 of which suffered from cerebral palsy and glaucoma (the 4th was an unintended conception and therefore was terminated on parental request), a couple decided to perform genetic analyses to discover if the cause could be found into inheritable conditions. No abnormality was found in chromosomal karyotype analysis (G-banding karyotyping, Fig. 1). Since the male partner was found to have oligoasthenospermia, further tests were planned (FISH), revealing the presence of a balanced translocation as t(9;13) (p24.3;q31.3). In addition, precise breakpoint regions of chromosomes 9 and 13 were characterized by the MicroSeq technique [6] using the husband's peripheral blood sample. The results showed that the breakpoint region of chromosome 9 in the carrier was 14,494,848–14,497,227 bp and that of chromosome 13 was 10,199,078–101,993,859 bp (Human reference Genome GRCh37/hg19). Thus, the husband was determined to be a carrier of the cryptic balanced translocation for t (9;13) (p24.3; q31.3).

### Genetic analysis

G-banding karyotyping of a culture of peripheral blood lymphocytes was performed according to the standard cytogenetic protocol. Fluorescence in situ hybridization (FISH) was performed on blood lymphocyte cultures according to published methods [12] using the probes RP11-54A12 (9q34.12-q34.13), RP11-569D9 (13q31.3), and RP11-31F19 (9p24.3).

Chromosome microdissection, amplification of the microdissected DNA, breakpoint mapping, and precise characterization of breakpoints for the translocated chromosomes for the carrier were performed as published [6].

The whole genome of the biopsied trophoblast cells was amplified using the REPLI-g Single Cell Kit (Qiagen, Hilden, Germany), followed by sequencing library construction using the gene sequencing library kit (Peking Jabrehoo Med Tech Co., Ltd., Beijing, China) and MiSeq (Illumina, San Diego, U.S.A.), NGS sequencing. PGT-A analysis of the data was performed by Peking Jabrehoo Med Tech., Ltd. Briefly, CNV analysis was performed as follows. After the low-quality bases and adaptors were removed, clean and high-quality reads were compared with the hg19 reference genome (University of California, Santa Cruz Genome Browser; genome.ucsc.edu/) using BWA (Burrows-Wheeler Alignment Tool, version 0.7.12-r1039). The relative copy numbers were calculated using the uniquely mapped reads against the reference dataset after removing the redundant reads using Picard software.

For euploid embryos detected by PGT-A, PGT-SR testing was performed for carrier embryo diagnosis as described [6].

### Preimplantation genetic testing of the embryos

**Fig. 2:**
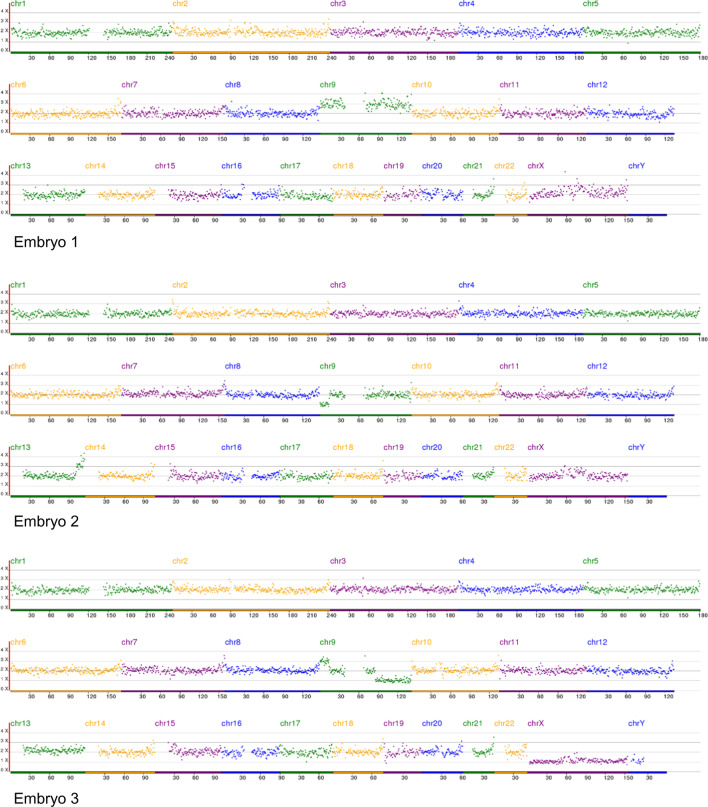

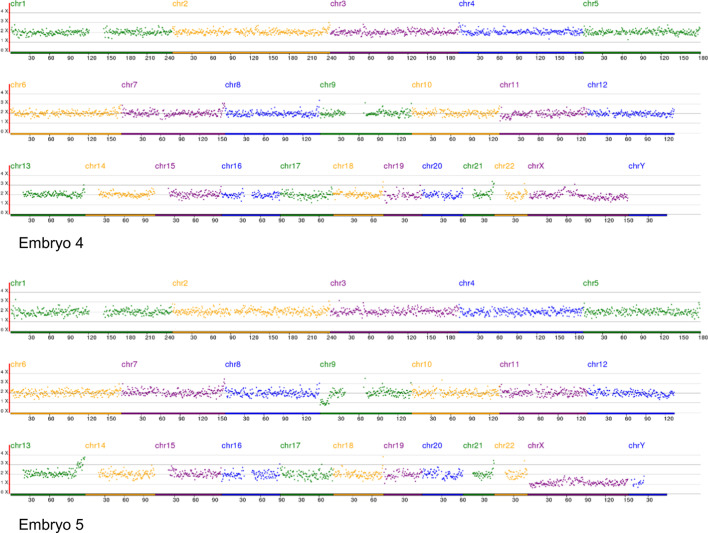
NGS-based PGT-A results of the 5 embryos. Embryos 1, 2, 3, and 5: aneuploid, embryo 4: euploid. Chr: chromosome

The couple in this study underwent two PGT cycles: two embryos (embryo 1–2) in the first cycle and three (embryo 3–5) in the second cycle. PGT-A testing of embryos 1–2 in the first cycle and embryos 3 and 5 showed that only embryo 4 was normal (Table 1, Fig. 2).

**Table 1:** PGT-A testing of the embryos of the family

Sample	PGT cycle	Karyotype	PGT-A result
Wife		Normal	–
Husband		t (9;13) (p24.3; q31.3)	–
Embryo 1	Cycle 1	+ (9)	Aneuploidy
Embryo 2		− (9)(p24.3-p23)(14.13 Mb)(10.095), + (13)(q33.1-q34)(13.06 Mb)(32.761)	Aneuploidy
Embryo 3	Cycle 2	+ (9)(p24.3-p23)(14.13 Mb)(29.949), − (9)(q21.32-q34.3)(55.86 Mb)(10.208)	Aneuploidy
Embryo 4		Normal karyotype	Balanced translocation carrier/normal
Embryo 5		− (9)(p24.3-p23)(14.13 Mb)(10.136), + (13)(q33.1-q34)(13.06 Mb)(30.849)	Aneuploidy

From the PGT-A results of the embryos and the karyotypes of the couple (Table 1), we can easily deduce the segregation mode of the gametes from the father: 3:1, 2:2 adjacent-1, chaotic, 2:2 alternate, and 2:2 adjacent-1 for the five embryos. In the five embryo-deriving gametes, 2:2 segregation was the main segregation mode, followed by 3:1, and no 4:0 segregation embryo was found, though the total number of the tested embryos was very small.

To determine whether embryo 4 was a normal embryo or a balanced translocation carrier, PGT-SR testing was performed. Linkage analysis using the informative SNPs determined from MicroSeq analysis showed that embryo 4 inherited the derivative chromosome from its father (Table 2) and was a balanced translocation carrier.

**Table 2:** PGT-SR testing of embryo 4

NO	Chr	ID (SNPs)	Ref	Alt	Husband	Wife	Embryo 4
*der(9)*							
S3	chr13	rs16958936	G	A	A/G	G/G	A/G
S4	chr13	rs9300676	G	T	T/G	T/T	T/G
S9	chr13	rs372606636	C	G	G/C	C/C	G/C
*der(13)*							
S10	chr9	rs117765092	G	C	C/G	G/G	C/G
S11	chr9	rs144416772	G	A	A/G	G/G	A/G
S12	chr9	rs9407550	C	A	A/C	C/C	A/C
S12	chr9	rs9407551	T	G	G/T	T/T	G/T
S13	chr9	rs1813529	C	T	T/C	C/C	T/C
S14	chr9	rs17221355	C	T	T/C	C/C	T/C
S15	chr9	rs7036035	T	C	C/T	T/T	C/T
S16	chr9	rs146845682	C	T	T/C	C/C	T/C

Ref: normal haplotype in the family; Alt: haplotype closely linked with the balanced translocation, red colour

### Clinical outcome

With the informed consent of the couple, the translocation embryo was thawed and transferred to obtain clinical pregnancy. Prenatal diagnosis with amniocentesis at 16 weeks of pregnancy confirmed that the foetal chromosomes were derivative. A baby girl was born by caesarean section at 38 weeks of pregnancy and was diagnosed as healthy by physical examination; she has continued to develop normally at six months’ follow-up.

## Discussion and conclusions

We described a case in which a healthy baby girl was born to a male carrier of a cryptic balanced translocation after PGT technique. As far as we know, this is the first report about successfully achieving live birth of a healthy baby through the PGT technique for a carrier of a cryptic balanced translocation.

The oligoasthenospermic male partner was found to be a carrier of the cryptic balanced translocation of t (9,13) (p24.3, q31.3) with FISH and MicroSeq techniques and evaded detection by G-banding karyotyping. The G-banding karyotype has a limit of detection of genomic imbalances above 5–10 Mb depending on the specific genomic region and can also miss rearrangements larger than 10 Mb [3] or even up to 18 Mb [13], especially aberrations close to telomeres since most terminal bands are G-negative [7]. In the case presented here, the translocated regions 9p24.3 at the terminal and 13q31.3 near the terminal were missed by the G-banding karyotyping and were detected with FISH. Additionally, the more precise breakpoint regions were determined with the MicroSeq technique, stressing the significance of higher resolution karyotyping methods in detecting rearrangements near the chromosomal terminals.

The preimplantation genetic testing technique has been exploited to help balanced translocation carriers give birth to healthy offspring [5, 14, 15], while only one case has been reported regarding assisted reproduction for cryptic balanced translocation carriers [10] with no successful pregnancy, which may be related to the high rate of chromosome abnormalities in embryos with translocations with terminal breakpoints [16]. In our study, only one balanced and no normal embryos were acquired in a total of 5 embryos biopsied in 2 PGT cycles, a very low incidence of normal or balanced embryos consistent with the other reported result [10]. A balanced translocation involving chromosomes with terminal breakpoints tends to form a quadrivalent with an open configuration in the first metaphase of meiosis, resulting in a higher proportion of unbalanced gametes [17]. Thus, the incidence of normal or balanced karyotypes in translocations with terminal breakpoints was significantly lower than that without terminal breakpoints (6.5% versus 14.4%, P = 0.001) [11], which requires testing a relatively large number of embryos to acquire at least 1–2 transferable normal or balanced embryos for cryptic balanced translocation carriers.

The segregation modes of gametes of balanced translocation carriers include alternate, adjacent-1, adjacent-2, 3:1, 4:0 and other chaotic segregation, and the last three modes are generally rare. For adjacent-2, the incidence is also low because homologous centromeres tend to separate during meiosis [18]. Inconsistent results were reported for segregation modes of gametes of balanced translocation involving chromosomes with terminal breakpoints: Ye et al. [11] reported a low incidence of alternate segregation and a relatively high incidence of adjacent-2 mode, while others reported a low frequency of or no adjacent-2 segregation [10, 19]. Though there were only five tested embryos in this study, no adjacent-2 segregation was found. The contradictory results may reflect the complexity of different methods and the specificity of chromosomes tested because translocations are usually unique in chromosomes and breakpoints in the genome.

The case we presented ended in a successful pregnancy and live born baby girl carrier for the same balanced translocation as her father, starting from 5 embryos, while the case described by McKenzie et al. [10] did not result in a viable pregnancy, starting from 18 embryos, which may reflect the higher sensitivity of the PGT/NGS detection technique for embryo aneuploidy than that of the FISH technique. Nonetheless, our methods have limitations. First, a relatively large number of embryos are needed for screening to acquire euploid embryos for live births because embryos with aberrations are more common than normal embryos. Second, more than one technique is needed to confirm the status of a cryptic balanced rearrangement, especially when the initial method misses the detection, which is tedious and burdensome for families plagued with failure to bear healthy offspring.

In summary, we report the first successful live birth of a healthy baby through the PGT technique for a couple in which the male partner is a carrier of the cryptic balanced translocation of t (9,13) (p24.3, q31.3), presumably causative for cerebral palsy and glaucoma. Our study showed that the PGT/NGS technique can effectively help families with a cryptic balanced translocation have healthy offspring.

## Data Availability

The datasets generated and analysed during the current study are not publicly available due to a concern to protect individual patient confidentiality, but are available from the corresponding authors on reasonable request.

## Abbreviations

FISH: Fluorescence in situ hybridization
PGT: Preimplantation genetic testing
PGT-A: Preimplantation genetic testing-aneuploidy
NGS: Next-generation sequencing
array CGH: Array comparative genomic hybridization
